# The Ketogenic Diet for Refractory Mental Illness: A Retrospective Analysis of 31 Inpatients

**DOI:** 10.3389/fpsyt.2022.951376

**Published:** 2022-07-06

**Authors:** Albert Danan, Eric C. Westman, Laura R. Saslow, Georgia Ede

**Affiliations:** ^1^Rangueil Faculty of Medicine, University of Toulouse, Toulouse, France; ^2^Department of Medicine, Duke University Medical Center, Durham, NC, United States; ^3^Department of Health Behavior and Biological Sciences, School of Nursing, University of Michigan, Ann Arbor, MI, United States; ^4^Independent Researcher, Northampton, MA, United States

**Keywords:** ketosis, schizophrenia, bipolar disorder, depression, mental disorders, diet therapy, inpatients

## Abstract

**Background and Hypothesis:**

The robust evidence base supporting the therapeutic benefit of ketogenic diets in epilepsy and other neurological conditions suggests this same metabolic approach may also benefit psychiatric conditions.

**Study Design:**

In this retrospective analysis of clinical care, 31 adults with severe, persistent mental illness (major depressive disorder, bipolar disorder, and schizoaffective disorder) whose symptoms were poorly controlled despite intensive psychiatric management were admitted to a psychiatric hospital and placed on a ketogenic diet restricted to a maximum of 20 grams of carbohydrate per day as an adjunct to conventional inpatient care. The duration of the intervention ranged from 6 to 248 days.

**Study Results:**

Three patients were unable to adhere to the diet for >14 days and were excluded from the final analysis. Among included participants, means and standard deviations (SDs) improved for the Hamilton Depression Rating Scale scores from 25.4 (6.3) to 7.7 (4.2), *P* < 0.001 and the Montgomery-Åsberg Depression Rating Scale from 29.6 (7.8) to 10.1 (6.5), *P* < 0.001. Among the 10 patients with schizoaffective illness, mean (SD) of the Positive and Negative Syndrome Scale (PANSS) scores improved from 91.4 (15.3) to 49.3 (6.9), *P* < 0.001. Significant improvements were also observed in metabolic health measures including weight, blood pressure, blood glucose, and triglycerides.

**Conclusions:**

The administration of a ketogenic diet in this semi-controlled setting to patients with treatment-refractory mental illness was feasible, well-tolerated, and associated with significant and substantial improvements in depression and psychosis symptoms and multiple markers of metabolic health.

## Introduction

Globally, an estimated 85 million people suffer from serious, persistent bipolar mood and psychotic illnesses (1), and at least 280 million (2) are thought to be afflicted with depressive illness. Yet even among those with access to modern professional care, meaningful improvement eludes many, and remission is rare. Nearly half of those receiving treatment for bipolar disorder continue to experience recurrent mood episodes (3). Across Europe, approximately 19% of those with depression are considered “treatment-resistant” (4). Worldwide, a mere 23% of those with schizophrenia respond well to antipsychotic medications (5), with symptom relief often coming at the expense of quality and length of life. Metabolic derangements such as hyperglycemia, hypertriglyceridemia, and weight gain are commonplace in those with bipolar disorder (6) as well as in those with schizophrenia (7), significantly increasing risk for obesity, type 2 diabetes, cardiovascular disease, and other chronic health conditions. Indeed, nearly two-thirds of patients initially hospitalized with acute psychosis develop obesity within 20 years of follow-up (8). Metabolic and other undesirable side effects drive approximately 74% of people to discontinue antipsychotic medicines within 18 months, contributing to high hospitalization and relapse rates (9).

These profound limitations of psychopharmacological treatments make the search for new approaches to mental illness of paramount importance. A compelling intervention attracting more attention in recent years is the ketogenic diet (KD), which restricts carbohydrate and induces lipolysis, generating circulating ketone bodies that serve as an adjunctive source of fuel for the brain, reducing its dependence on glucose (10).

Although the study of KDs for the treatment of psychiatric illnesses is in its infancy, the implementation of KDs in neurological illnesses dates back a century, when they first proved useful in the management of epilepsy (11). The now robust evidence base supporting the application of the KD to epilepsy and a growing number of other challenging neurological conditions (12) suggests this same metabolic approach may also benefit psychiatric conditions (13). For example, it is well established that epilepsy and bipolar illness share many neurochemical underpinnings, and this overlap is clinically supported by the fact that many of the same molecules prescribed to control seizures are also prescribed to stabilize mood (14). Indeed, the line separating brain illnesses considered neurological in nature from those considered psychiatric in nature may be more rhetorical than biological (15), as both categories of disease originate within the same organ and display many biochemical similarities, including dysregulation of neurotransmitter systems, destabilization of neural networks, neuroinflammation, excessive oxidative stress, impaired neuroplasticity, mitochondrial dysfunction, and disturbed cerebral glucose metabolism (16–18).

However, as rigorous clinical trial evidence is not yet available in this field, it remains unclear to what extent serious mental illnesses may benefit from a metabolic approach. Therefore, to the isolated case reports of individuals with major depressive illness (19), bipolar illness (20, 21), and psychotic illness (22) who have benefited from a KD, we add this case series of 31 patients with treatment-refractory mental illness treated with a KD in a semi-controlled hospital setting.

## Context

Dr. Danan, the first author, has been a practicing psychiatrist in Toulouse, France for 35 years. The population he serves is comprised primarily of people of French and North African descent with serious, persistent mental illness, many of whom who also suffer from metabolic illnesses such as obesity, hypertension, and type 2 diabetes. Despite intensive outpatient psychopharmacologic and psychotherapeutic management, most of these individuals require frequent hospitalization and are unable to work due to psychiatric disability. After witnessing marked improvement in medication-refractory seizures and autism behaviors in a family member within several weeks of having adopted a KD, Dr. Danan became interested in the potential of the KD to improve the psychiatric and metabolic status of his most treatment-resistant patients, regardless of diagnosis. He created a metabolic psychiatry treatment program within the Clinique du Castelviel, a 129-bed general psychiatric hospital in Castelmarou, France where patients with chronic mental illness who had exhausted standard psychiatric therapies could attempt a KD in a supportive, medically supervised environment.

## Materials and Methods

This is a retrospective analysis of hospitalized adults with serious and persistent mental illness who were provided a KD in lieu of the standard hospital menu. Between May 2019 and April 2020, 31 adults whose chronic psychiatric symptoms were poorly controlled despite intensive psychopharmacological management were admitted to the Clinique du Castelviel and placed on a KD under the supervision of their treating psychiatrist, Dr. Danan. This treatment program was approved by Clinique du Castelviel administration and ethics review.

### Participants

Participants were uncompensated volunteers selected by Dr. Danan from his outpatient psychiatric practice. Informed consent was obtained in every case. Eligibility criteria were failure to respond adequately to conventional psychiatric care and willingness to try a KD. Exclusion criteria were anorexia nervosa, BMI below 18.5 kg/m^2^, pregnancy, breastfeeding, and contraindicated medical conditions (23). Primary psychiatric diagnoses were bipolar disorder type two (*n* = 13), schizoaffective disorder (*n* = 12), and major depressive disorder (*n* = 7). All participants had at least one indicator of poor metabolic health, such as overweight, obesity, hypertension, and/or elevated fasting blood glucose. Most participants had been in Dr. Danan's care for many years (mean [SD] 10 [7] years, range 5 months to 30 years), and all had been psychiatrically hospitalized in the past under his supervision one or more times either at this same facility or a similar affiliated facility with minimal clinical improvement. None of the participants had ever attempted to follow a low-carbohydrate diet before.

Of 31 patients, 22 were voluntarily admitted for the express purpose of initiating the KD in a monitored setting. The remaining 9 were initially admitted for conventional care but later agreed to the KD because non-dietary interventions proved ineffective. All 31 were taking psychotropic medication at the time of KD initiation.

In addition to the KD protocol, which was the cornerstone of their treatment plan, participants also received the usual care available to all patients admitted to this unit. Hospital staff was comprised of 8 psychiatrists, 3 psychologists, 2 general medical practitioners, 2 social workers, an occupational therapist, an exercise instructor, and a dietitian. Illnesses typically treated at this facility included mood disorders, schizophrenia, substance use disorders, and eating disorders. Participants resided on the inpatient unit 6 days per week but were free to leave the hospital on weekends for up to 36 consecutive hours.

### Statistical Analysis

Means and standard deviations were calculated for continuous variables; frequency distributions were calculated for nominal and ordinal variables. We used paired *t*-tests when the pre-post differences had a normal distribution and Wilcoxon signed-rank tests when the differences were skewed, using SPSS 28.0.1.1.

### Interventions

The KD protocol employed was adapted from that used by Dr. Westman in clinical trials at Duke University (24); see Supplementary Figure 1: Ketogenic Diet Protocol for details and a sample meal plan. Briefly, carbohydrate intake was restricted to a maximum of 20 total grams per day (approximately 5% of daily calories), exclusively from vegetables, nuts, lemon juice, and small amounts of dark chocolate. Protein comprised 15–20% of daily calories and was sourced from meat, seafood, poultry, dairy products, eggs, and nuts. Fat comprised 75–80% of the diet; added fats permitted were olive oil, coconut oil, butter, mayonnaise, and sour cream.

Participants were provided with 3 protocol-compliant meals per day (prepared by the hospital dietitian to ensure sufficient protein and calories), 1 protocol-compliant snack box per day (formulated by Dr. Danan, purchased by participants, and dispensed daily by Dr. Danan), and a list of approved foods to adhere to at all times. Participants also received once-daily supplementation of fish oil (250 mg: 18% EPA, 12% DHA), magnesium oxide (300 mg), copper (1 mg), vitamin B1 (1.1 mg), vitamin B5 (6 mg), vitamin B6 (3.4 mg), vitamin B12 (2.5 mg), and vitamin C (330 mg).

Dr. Danan met individually with each participant 6 days per week to monitor clinical progress and provide dietary education and support. Dietary adherence was estimated using information gathered from these frequent physician interviews, participants' daily food journals, and nursing observations. Adherence was characterized as excellent in those who successfully limited carbohydrate intake to a maximum of 20 grams per day at least 6 days per week, good in those who met this goal at least 5 days per week, and fair in those who met this goal at least 4 days per week. Urine acetoacetate was measured at least once per participant during the intervention period. Metabolic monitoring including blood tests, blood pressure, and body weight was conducted on day 0 of the KD and again on the final day of the KD, just prior to hospital discharge.

Admission date, hospitalization length, KD duration, and non-dietary aspects of care varied depending on clinical circumstances. Medications were adjusted based on clinical judgment.

### Main Outcome Measures

Main outcome measures determined prior to the intervention were change in depression symptoms as measured by the Hamilton Depression Rating Scale (HAM-D) (25) and the Montgomery-Åsberg Depression Rating Scale (MADRS) (26), and change in psychosis symptoms as measured by the Positive and Negative Syndrome Scale (PANSS) (27, 28).

Secondary outcomes of interest included medical and psychiatric safety, effect on metabolic biomarkers, change in medication requirements, and change in illness severity, assessed using the Clinical Global Impressions Scale (CGI-S) (29, 30).

## Results

### Patient Characteristics

Three of 31 patients (10%) were unable to follow a KD for >14 days and were excluded from the final analysis due to lack of outcome data; this case series is therefore comprised of 28 hospitalized adults [mean (SD) age, 50 (11.3) years, range 27–73 years; 71% female]. Mean (SD) hospitalization length was 85.4 (76.8) days (range 16–270 days) and mean (SD) duration of KD was 59.1 (49.6) days (range 15–248 days).

### Dietary Adherence

Regarding the 3 excluded patients mentioned above: 1 discontinued the KD citing aversion to dietary fat, 1 cited lack of family and financial support, and 1 cited financial hardship, aversion to dietary fat, and dislike for the restrictiveness of the plan.

Among the included 28 patients who followed the diet for more than 2 weeks, urine ketone measurements were obtained once during the intervention and were positive in 18 of 28 patients (64%). Dietary adherence was characterized as excellent in 11 patients (39%), good in 12 patients (43%), and fair in 5 patients (18%).

Changes in the mental health measures described below are presented in Table 1. Noticeable improvements in mood and psychotic symptoms were observed in all 28 patients (100%) during the intervention, typically within 3 weeks or less of initiating the KD.

**Table 1:** Changes in mental health measures.

**Clinical scale**	***n* ^a^**	**Pre-KD, mean (SD)**	**Post-KD, mean (SD)**	**Change**	**Percent change**	**Cohen's d**	***P*-value**
**Overall**							
HAM-D	23	25.4 (6.3)	7.7 (4.2)	−17.7 (5.7)	−69.2 (14.2)	3.1	<0.001
MADRS	21	29.6 (7.8)	10.1 (6.5)	−19.5 (5.4)	−67.4 (15.0)	3.6	<0.001
PANSS	10	91.4 (15.3)	49.3 (6.9)	−42.1 (12.1)	−45.4 (7.1)	3.5	<0.001
CGI-S	27	4.9 (1.2)	2.0 (1.1)	−2.9 (0.8)	−60.4 (14.8)	3.8	<0.001
**Primary diagnosis: Bipolar disorder**							
HAM-D	12	24.9 (7.3)	9.2 (5.1)	−15.8 (6.5)	−62.6 (16.1)	2.4	<0.001
MADRS	12	29.9 (8.5)	11.8 (7.6)	−18.2 (6.2)	−62.1 (16.7)	2.9	<0.001
PANSS	12	n/a	n/a	n/a	n/a	n/a	n/a
CGI-S	12	4.8 (1.2)	2.0 (1.3)	−2.8 (0.9)	−60.7 (18.4)	3.0	0.002
**Primary diagnosis: Major depression**							
HAM-D	6	24.0 (3.8)	5.5 (2.6)	−18.5 (2.3)	−77.8 (7.9)	7.9	0.026
MADRS	6	27.3 (5.3)	7.2 (3.2)	−20.2 (2.2)	−75.0 (7.9)	9.0	<0.001
PANSS	6	n/a	n/a	n/a	n/a	n/a	n/a
CGI-S	6	4.3 (0.5)	1.3 (0.5)	−0.3 (0.5)	−70.0 (7.7)	n/a^b^	0.014
**Primary diagnosis: Schizoaffective disorder**							
HAM-D	5	28.2 (6.1)	7.0 (1.6)	−21.2 (5.7)	−74.7 (5.8)	3.7	0.001
MADRS	3	32.7 (10.4)	9.3 (5.7)	−23.3 (6.1)	−73.3 (12.3)	3.8	0.022
PANSS	10	91.4 (15.3)	49.3 (6.9)	−42.1 (12.1)	−45.4 (7.1)	3.5	<0.001
CGI-S	9	5.4 (1.3)	2.6 (0.9)	−2.9 (0.8)	−53.7 (9.6)	3.7	0.007

*HAM-D is a 17-item clinician-administered diagnostic questionnaire rated on a scale of 0 to 52, with ≤ 7 indicating no depression and ≥24 indicating severe depression. The MADRS is rated on a scale of 0 to 60, with ≤ 6 indicating no depression and ≥35 indicating severe depression. PANSS is rated on a scale of 30 to 210, with 30 considered the least severe and ≥116 considered severely ill. CGI-S is rated on a scale of 1 to 7, with 1 indicating normal and 7 indicating extreme illness.*

a *As this is a retrospective analysis, HAM-D, MADRS, and CGI-S scores were not available for all 28 participants.*

b *Not calculated by SPSS*.

### Schizophrenia Symptoms

Following the KD intervention, all 10 (100%) patients with a primary diagnosis of schizoaffective disorder exhibited improvement in PANSS scores, with the mean (SD) PANSS score falling from 91.4 (15.3) to 49.3 (6.9), *P* < 0.001, Cohen's *d* = *3.5*. A reduction of 16.5 or more points in the PANSS score is considered the minimal clinically important difference (31) and was achieved in 10/10 (100%) patients.

### Depression Symptoms

The HAM-D was administered to 23 of 28 patients, including all 18 with a primary diagnosis of a non-psychotic illness. Following the KD intervention, all 23 (100%) patients exhibited improvement in HAM-D scores, with the mean (SD) HAM-D score falling from 25.4 (6.3) to 7.7 (4.2), *P* < 0.001, Cohen's *d* = 3.1. The MADRS was administered to 21 of 28 patients, including all 18 with a primary diagnosis of a non-psychotic illness. Following the KD intervention, all 21 (100%) exhibited improvement in MADRS scores, with the mean (SD) MADRS score falling from 29.6 (7.8) to 10.1 (6.5), *P* < 0.001, Cohen's *d* = 3.6.

A reduction of 4 or more points in the HAM-D is considered the minimal clinically important difference and was achieved by all 22 patients in whom HAM-D scores were assessed, regardless of diagnosis. A reduction of 7 or more points, which is considered substantially clinically important, was achieved by 21/22 patients (95%) (32).

A reduction of at least 6 points in the MADRS is considered the minimal clinically important difference and was achieved by all 21 patients (100%) in whom MADRS scores were assessed, regardless of diagnosis.

### CGI-S

**Figure 1:**
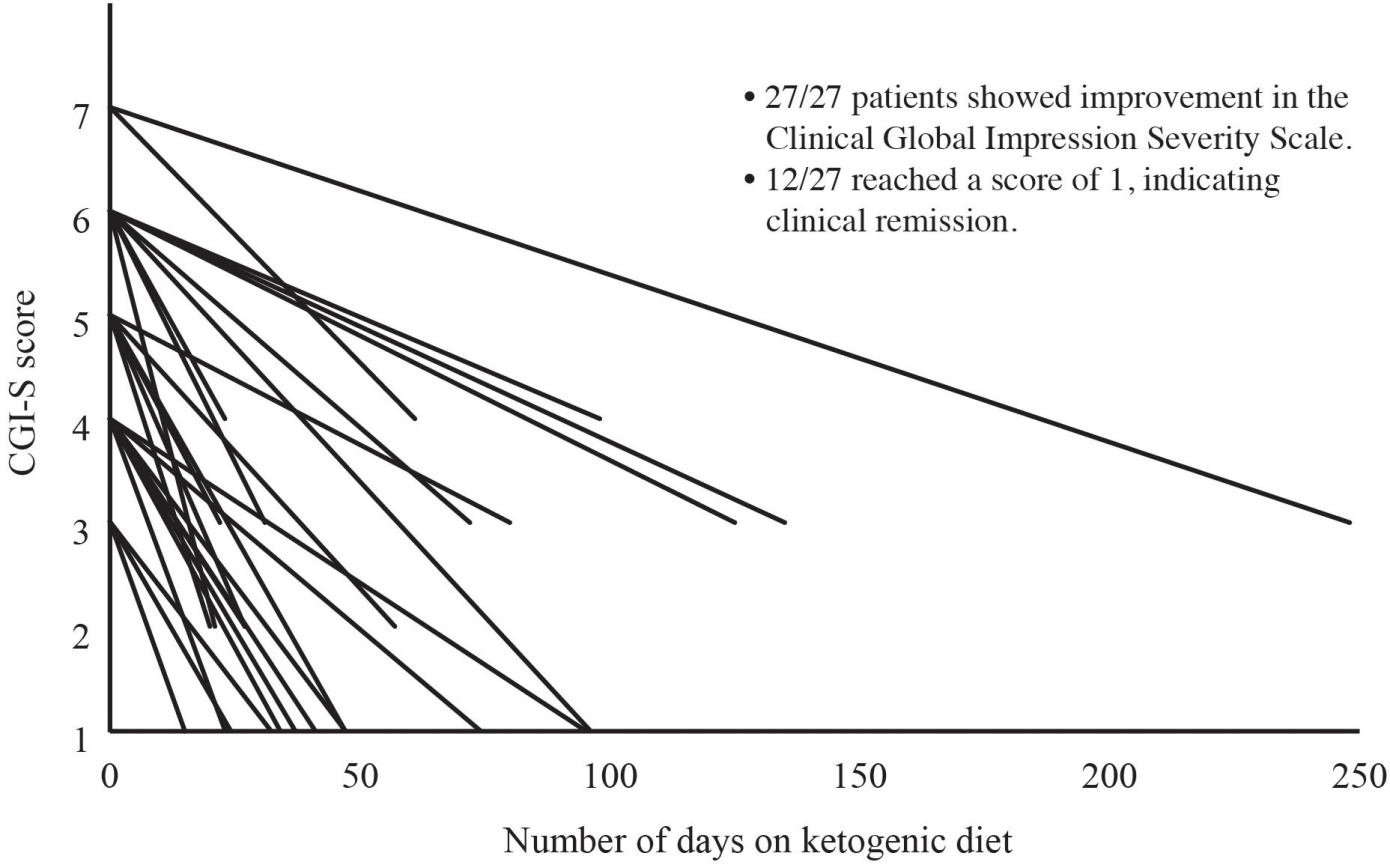
Change in Clinical Global Impressions Severity Scale (CGI-S) Over Time. Severity of illness was assessed in 27 of 28 patients using the CGI-S. The CGI-S is rated on a scale of 1 to 7, with 1 indicating normal and 7 indicating extreme illness. Following the KD intervention, CGI-S had improved in all 27 patients, with 12 of 27 (44%) achieving a CGI-S of 1 (clinical remission).

Severity of illness was assessed in 27 of 28 patients using the CGI-S. Following the KD intervention, mean (SD) CGI-S improved from 4.9 (1.2) to 2.0 (1.1), Cohen's *d* = *3.8*. As this change violated the assumption of normality, we conducted a Wilcoxon Signed-Ranks Test that indicated that the change was statistically significantly different, Z = −4.65, *P* < 0.001 (see Figure 1), with 12 of 28 patients (43%) achieving clinical remission. A reduction of 1 point on the CGI-S is considered the minimal clinically important difference (33). All patients achieved a reduction of at least 2 points on the CGI-S, regardless of diagnosis (Cohen's d = 3.8).

### Medication Changes

**Figure 2:**
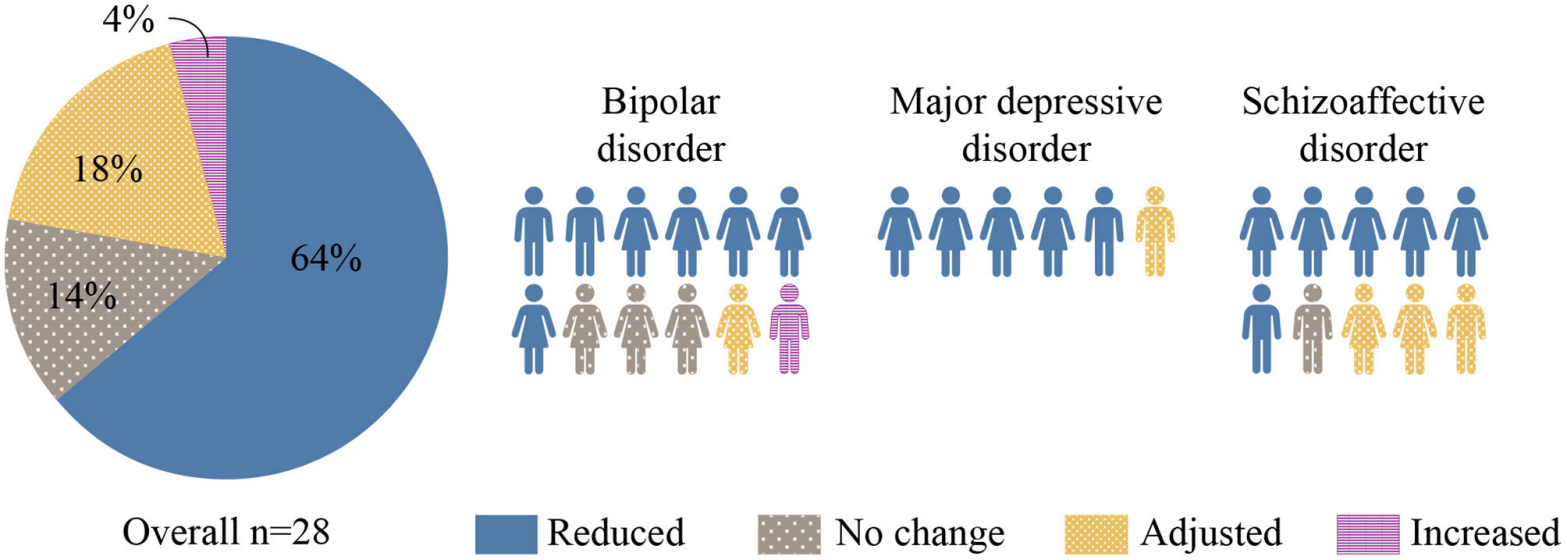
Changes in Psychotropic Medication. Changes in the number and/or dosage of psychotropic medications associated with KD intervention are represented in this figure. The majority (64%) of participants were discharged on less medication.

Prior to the intervention, the mean (SD) number of psychotropics taken per patient was 5.3 (2.0) with 25 of 28 (89%) patients taking at least one antipsychotic medication. By the end of the intervention, the number and/or dosage of psychotropic medications had been reduced in 18 of 28 (64%) patients (see Figure 2). Among the 7 patients who were also taking non-psychotropic medications, the number and/or dosage of those medications was reduced in 5 of 7 patients (71%). Somatic medications reduced and/or discontinued were insulin, metformin, atorvastatin, gliclazide, and ticagrelor.

### Metabolic Health Measures

Metabolic health measures are detailed in Table 2. Prior to the intervention, mean (SD) body weight was 198.5 (42.1) lbs (range 145.7–310.9 lbs) and mean (SD) BMI was 31.9 (6.7) kg/m^2^ (range 23.0–51.2 kg/m^2^). Initial BMI lay in the normal range (18.5–24.9 kg/m^2^) for 3/28 (10.7%) patients, in the overweight range (25.0–29.9 kg/m^2^) for 7/28 (25.0%) patients, and in the obese range (≥30.0 kg/m^2^) for 18/28 (64.3%) patients. At the conclusion of the intervention, all but one patient (27 of 28; 96.4%) had lost weight. Of note, 24 of the 25 (96%) patients who were taking antipsychotic medications lost weight, and 12 of those 25 (48%) achieved clinically significant weight loss [defined as ≥5% reduction in body weight (34)]. Overall, weight change [mean (SD), −10.8 (−7.1) lbs]; and BMI change [mean (SD), −1.7 (1.2) kg/m^2^] were both significant (*P* < 0.001), as were reductions in fasting blood glucose, hemoglobin A1c, systolic and diastolic blood pressure, and gamma-glutamyl transferase (GGT). There were also significant overall reductions in alanine aminotransferase (ALT), aspartate aminotransferase (AST), total cholesterol, and triglycerides (TGs). Among the 14 of 28 (50%) patients who initially met criteria for hypertriglyceridemia (TG > 150 mg/dl), marked reductions of 100 mg/dl or more were seen in 7 (50%), with 5 patients (36%) no longer meeting criteria for hypertriglyceridemia following the intervention. C-reactive protein levels were also measured but due to several instances of infection, values were deemed unreliable.

**Table 2:** Metabolic health measures.

**Clinical value**	**n^a^**	**Pre-KD, mean (SD)**	**Post-KD, mean (SD)**	**Change**	**Percent change**	***P*-value**
**Overall**						
Weight, lbs	28	198.5 (42.1)	187.7 (38.5)	−10.8 (7.1)	−5.3 (3.1)	<0.001
BMI, kg/m^2^	28	31.9 (6.7)	30.1 (6.1)	−1.7 (1.2)	−5.3 (3.1)	<0.001
SBP, mmHg	26	134.5 (15.0)	123.4 (11.3)	−11.1 (12.6)	−7.6 (9.6)	<0.001
DBP, mmHg	26	85.7 (11.8)	78.7 (9.0)	−7.0 (8.5)	−7.4 (9.6)	<0.001
FBG, mg/dL	27	104.7 (26.7)	93.4 (14.2)	−11.2 (19.3)	−8.1 (14.9)	0.002
HbA1c, %	24	5.9 (0.7)	5.6 (0.5)	−0.2 (0.5)	−3.5 (6.5)	0.003
Trig, mg/dL	28	204.0 (194.7)	137.5 (69.5)	−66.5 (149.5)	−14.8 (35.4)	0.003
HDL-C, mg/dL	27	50.1 (11.6)	54.0 (17.0)	3.9 (12.9)	8.4 (26.6)	0.125
LDL-C, mg/dL	28	141.4 (40.6)	131.5 (29.7)	−9.9 (37.2)	−0.5 (36.8)	0.171
Total Chol, mg/dL	28	226.7 (44.6)	210.4 (31.0)	−16.3 (37.0)	−4.4 (21.1)	0.027
ALT, u/L	25	43.5 (74.2)	32.8 (28.6)	−10.7 (56.2)	−1.4 (55.7)	0.036
AST, u/L	26	27.7 (24.1)	22.5 (11.5)	−5.1 (17.2)	−9.0 (40.1)	0.009
GGT, u/L	26	64.4 (117.6)	36.4 (38.6)	−28.0 (81.2)	−21.0 (30.9)	0.002
TSH, μU/mL	24	2.1 (2.7)	1.8 (1.2)	−0.2 (2.3)	28.2 (95.1)	0.831
UA, mg/dL	24	56.3 (23.5)	53.6 (18.3)	−2.6 (15.0)	−0.3 (22.0)	0.749
**Primary diagnosis: Bipolar disorder**						
Weight, lbs	12	191.7 (32.9)	182.3 (31.3)	−9.4 (5.9)	4.9 (2.9)	<0.001
BMI, kg/m^2^	12	31.0 (5.0)	29.5 (4.7)	−1.5 (1.0)	−4.9 (2.9)	<0.001
SBP, mmHg	12	131.2 (12.6)	120.9 (14.0)	−10.3 (11.6)	−7.6 (9.1)	0.011
DBP, mmHg	12	83.1 (13.0)	75.1 (9.9)	−8.0 (10.1)	−8.7 (11.1)	0.019
FBG, mg/dL	12	101.0 (19.7)	90.8 (15.5)	−10.3 (9.5)	−9.4 (8.9)	0.003
HbA1c, %	11	6.1 (0.7)	5.8 (0.5)	−0.3 (0.6)	−4.6 (8.1)	0.111
Trig, mg/dL	12	284.1 (267.2)	167.2 (86.4)	−116.9 (215.0)	−25.7 (32.3)	0.012
HDL-C, mg/dL	11	50.0 (12.9)	52.5 (19.7)	2.5 (12.6)	4.0 (27.4)	0.929
LDL-C, mg/dL	12	144.2 (40.6)	121.4 (35.2)	−22.8 (30.0)	−14.6 (19.1)	0.024
Total Chol, mg/dL	12	241.5 (42.9)	207.6 (36.6)	−33.9 (31.1)	−13.3 (11.9)	0.003
ALT, u/L	9	62.6 (110.6)	36.0 (31.7)	−26.6 (79.9)	−11.1 (29.3)	0.153
AST, u/L	11	34.2 (35.4)	23.0 (13.0)	−11.2 (22.8)	−21.0 (15.3)	0.003
GGT, u/L	12	100.5 (168.7)	46.8 (54.4)	−53.7 (115.6)	−34.3 (17.5)	0.002
TSH, μU/mL	10	1.7 (0.9)	2.0 (1.3)	0.4 (1.3)	45.4 (111.7)	0.414
UA, mg/dL	11	65.7 (23.7)	60.7 (15.8)	−5.0 (19.5)	−1.7 (26.3)	0.414
**Primary diagnosis: Major depression**						
Weight, lbs	6	213.7 (56.1)	203.1 (50.0)	−10.6 (7.1)	−4.6 (2.1)	0.015
BMI, kg/m^2^	6	34.9 (8.0)	33.2 (7.1)	−1.7 (1.1)	−4.6 (2.1)	0.014
SBP, mmHg	5	144.0 (18.2)	129.4 (11.2)	−14.6 (10.4)	−9.6 (7.1)	0.035
DBP, mmHg	5	95.4 (12.4)	86.2 (8.6)	−9.2 (5.1)	−9.3 (4.4)	0.015
FBG, mg/dL	5	84.6 (14.7)	91.8 (10.8)	7.2 (11.6)	9.9 (14.9)	0.238
HbA1c, %	5	5.9 (0.6)	5.8 (0.5)	−0.1 (0.4)	−2.1 (7.0)	0.481
Trig, mg/dL	6	137.3 (98.3)	126.5 (53.8)	−10.8 (46.0)	6.0 (27.0)	0.917
HDL-C, mg/dL	6	48.2 (14.4)	52.7 (12.5)	4.5 (3.1)	11.1 (9.1)	0.016
LDL-C, mg/dL	6	141.3 (28.4)	155.3 (22.1)	14.0 (45.4)	15.6 (39.6)	0.484
Total Chol, mg/dL	6	217.7 (20.6)	219.2 (20.6)	1.5 (20.5)	1.0 (8.8)	0.500
ALT, u/L	5	32.8 (20.9)	43.6 (42.6)	10.8 (38.4)	31.2 (113.5)	0.686
AST, u/L	5	26.4 (12.3)	30.6 (13.4)	4.2 (16.8)	32.0 (70.8)	0.605
GGT, u/L	5	32.0 (19.1)	30.6 (13.3)	−1.4 (18.8)	7.1 (37.9)	0.876
TSH, μU/mL	5	1.4 (0.6)	1.9 (0.4)	0.5 (1.0)	70.6 (115.9)	0.317
UA, mg/dL	4	46.5 (11.5)	44.0 (17.0)	−2.5 (14.5)	−4.8 (30.9)	0.754
**Primary diagnosis: Schizoaffective disorder**						
Weight, lbs	10	197.6 (45.2)	185.0 (40.8)	−12.6 (8.5)	−6.2 (3.9)	0.001
BMI, kg/m^2^	10	31.1 (7.8)	29.1 (7.0)	−2.0 (1.4)	−6.2 (3.8)	0.001
SBP, mmHg	9	133.8 (15.7)	123.4 (5.8)	−10.3 (15.7)	−6.5 (12.0)	0.084
DBP, mmHg	9	83.7 (6.9)	79.2 (5.2)	−4.4 (7.9)	−4.78 (9.9)	0.171
FBG, mg/dL	10	119.1 (31.8)	97.5 (14.5)	−12.6 (24.6)	−15.5 (14.1)	0.007
HbA1c, %	8	5.5 (0.5)	5.3 (0.5)	−0.2 (0.2)	−2.7 (3.5)	0.064
Trig, mg/dL	10	147.9 (78.5)	108.5 (39.8)	−39.4 (55.5)	−14.2 (40.4)	0.052
HDL-C, mg/dL	10	51.4 (9.1)	56.6 (17.4)	5.2 (17.0)	11.6 (33.4)	0.360
LDL-C, mg/dL	10	138.0 (49.6)	129.2 (18.2)	−8.8 (36.2)	6.7 (47.4)	0.462
Total Chol, mg/dL	10	214.4 (54.6)	208.6 (30.9)	−5.8 (44.0)	3.1 (30.5)	0.687
ALT, u/L	9	26.0 (8.9)	22.9 (9.7)	−3.1 (7.6)	−7.8 (30.8)	0.257
AST, u/L	10	21.1 (7.5)	18.0 (6.5)	−3.2 (4.4)	−16.2 (28.3)	0.047
GGT, u/L	9	34.2 (16.4)	25.8 (14.1)	−8.4 (13.5)	−18.8 (32.9)	0.098
TSH, μU/mL	9	2.8 (4.3)	1.5 (1.4)	−1.3 (3.4)	−14.4 (41.2)	0.123
UA, mg/dL	9	49.0 (24.7)	49.2 (20.2)	0.2 (8.7)	3.5 (11.8)	0.528

*ALT, alanine transferase; AST, aspartate transferase; BMI, body mass index; DBP, diastolic blood pressure; FBG, fasting blood glucose; GGT, gamma glutaryl transferase; HbA1c, hemoglobin A1c; HDL-C, high density lipoprotein cholesterol; LDL-C, low density lipoprotein cholesterol; SBP, systolic blood pressure; Total Chol, total cholesterol; Trig, serum triglycerides; TSH, thyroid stimulating hormone; UA, uric acid.*

a *As this is a retrospective analysis, some values were not available for all 28 participants*.

### Ketogenic Diet Tolerability

Most patients initially experienced one or more symptoms commonly reported during early keto-adaptation (35), such as headache, insomnia, irritability, excitation, dizziness, and carbohydrate cravings. These were mild, required no special medical or psychiatric management, and resolved within 2 weeks or less. Beyond this initial transition period, the KD was psychiatrically well tolerated by all patients, and 27 of 31 (87%) experienced no problematic somatic side effects. Two (excluded) patients cited fat intolerance, and 2 (included) patients experienced diarrhea and/or vomiting which resolved within 4 weeks. A fifth patient developed gastroenteritis during week 5 of the intervention and discontinued the diet, but later resumed the KD without ill effects, suggesting gastroenteritis was unlikely to have been KD related.

### Post-Hospitalization Dietary Adherence

In the months following hospital discharge, 13 of the 28 included patients (46%) reported good adherence to the KD at home, 5 (18%) reported partial adherence, 6 (21%) discontinued the diet, 1 discontinued then later resumed, and 3 were lost to follow-up. Those who elected to continue the KD after discharge did so to maintain or improve upon the psychiatric and metabolic benefits experienced during hospitalization. Reasons for discontinuation included cost, difficulty preparing meals, restrictiveness, and low motivation.

## Discussion

This iteration of a KD was safe, feasible to administer in an inpatient setting, well tolerated by most patients, and associated with substantial and statistically significant improvements in symptoms of depression and psychosis not observed during previous hospitalizations. Effect sizes were large (Cohen's d > 0.8) (36) across all mental health outcome measures in all subgroups, and were very large among those with a primary diagnosis of major depression. Given that the interventions implemented during this hospitalization differed only in the addition of the KD to usual care, we believe it is likely that the KD contributed considerably to these unprecedented mental health improvements, particularly in the 79% of patients whose psychotropic medications were either reduced or unchanged. The notable improvements in multiple markers of metabolic health including body weight, blood pressure, blood glucose, and triglycerides observed in this series would be difficult to ascribe to any aspect of the hospitalization other than the KD, which is known to facilitate these healthy changes (37).

### Historical and Scientific Context

A recent non-randomized study of 262 outpatients with type 2 diabetes treated with a KD detected significant improvement in mood. This cohort included 36 patients with mild clinical depression, more than half of whom no longer met criteria for depression at week 10 (38).

To the best of our knowledge, this case series represents the first exploration of the use of the KD in hospitalized patients with bipolar illness or severe depressive illness, and only the second exploration of the use of the KD in hospitalized patients with psychotic illness—the first having been published in 1965 (39). In that pilot study, a KD was administered to 10 women with treatment-refractory schizophrenia, resulting in statistically significant improvements in mean symptom scores after 2 weeks. Since that time, a number of isolated case reports describing people with mental illness who have benefited substantially from KDs have been documented; the first of these was published by one of us in 2009 (40) and offers an example of long-lasting resolution of psychotic symptoms and the ability to completely discontinue antipsychotic medication.

### Biological Plausibility

The biological plausibility that KDs may be of therapeutic benefit in major depression, bipolar illness, and schizophrenia is strongly supported by the scientific literature (18, 41–44).

#### Major Depression

Inflammation is implicated in all three conditions but has been most extensively studied in depression. Inflammation plays a role in the development and course of many cases of clinical depression and is associated with poor response to antidepressant medications (45, 46). The KD has been shown to reduce inflammation via complex influences on both central and peripheral immunoregulatory pathways (47–50). The KD also influences multiple neurotransmitter systems involved in depression, including the dopaminergic, serotonergic, glutamatergic, and GABAergic systems (51).

#### Bipolar Disorder

Among those with bipolar illness, there is a higher prevalence of impaired glucose metabolism even in drug-naïve individuals (52). Calkin (53) found that those with insulin resistance or type 2 diabetes are more likely to experience rapid mood cycling, less likely to respond to lithium, and more likely to suffer a more progressive disease course. Proposed mechanisms by which glucose and insulin dysregulation may dysregulate mood include damaging oxidative stress which, in turn, could impair mitochondrial function (16). Napolitano et al. (54) recently discovered that the KD can increase brain levels of glutathione, a ubiquitous intracellular antioxidant key to buffering oxidative stress. Campbell and Campbell (55) hypothesize that the KD might help ameliorate symptoms of bipolar illness by shifting the brain's primary fuel source from glucose to ketone bodies, thereby bypassing existing mitochondrial defects and reducing further mitochondrial injury. Calkin et al. (56) have proposed that insulin resistance, via inflammatory damage to endothelial cells, can compromise the integrity of the blood-brain barrier (BBB) in people with bipolar illness. Interestingly, disruption of the tight junctions critical to BBB structure and function has been observed not only in bipolar illness but also in major depression and schizophrenia (57).

#### Schizophrenia

Hyperinsulinemia, insulin resistance, and impaired glucose metabolism are more common in treatment-naïve individuals experiencing first-episode psychosis than in the general population (58). While this association alone is insufficient to support a causal relationship between metabolic dysregulation and psychotic symptoms, several cases of acute hyperglycemia associated with transient psychotic symptoms in patients with type 1 and type 2 diabetes have been reported (59). Pathophysiological features of schizophrenia shown in pre-clinical studies by Sarnyai et al. (44) to improve in response to a KD include N-methyl-D-aspartate (NMDA) receptor hypofunction, sensory gating deficits, and glutamate excitotoxicity.

KDs also help rebalance neurotransmitter systems, (16) stabilize neural networks (60), improve neuroplasticity (61), and bridge the energy gap resulting from the cerebral glucose hypometabolism associated with major depression, bipolar illness, and schizophrenia (17).

### Strengths and Limitations

Unique strengths of this series include the diversity of psychiatric diagnoses and the relatively large number of patients exposed to the same intervention in the same semi-controlled clinical setting. It was with these elements in mind that we chose not to report these patient outcomes as individual case studies. The provision of education, monitoring, and support by a psychiatrist who had trusting therapeutic alliances with his patients prior to the intervention seemed to contribute to patients' openness to the intervention and likely improved dietary adherence and honesty about transgressions, although this special treatment context makes it unclear whether these same outcomes would be possible under different circumstances.

Neither the treating psychiatrist responsible for assessing outcomes nor the patients themselves were blinded to the intervention, therefore there is a risk that impressions of clinical progress may have been biased. While patients may have benefited simply from having been hospitalized, a strength of this cohort is that all patients had previously been hospitalized under Dr. Danan's care at least once (and in many cases, multiple times) at either this same facility or a sister facility where all non-dietary aspects of care were very similar, yet Dr. Danan reports never having observed this degree of clinical improvement in these patients before. These patients therefore might be considered to have served as their own historical comparison group, although mental health outcomes were not formally measured during previous hospitalizations.

As with any intervention in which ad libitum dietary patterns are replaced with a structured dietary pattern of interest, multiple dietary variables were manipulated, therefore even if it were possible to assign clinical benefits to the KD, it would still be difficult to determine which aspect(s) of the KD may be responsible for those benefits. For example, this iteration of the KD was not only low in carbohydrate—it was also grain-free, very low in processed foods, and supplemented with micronutrients. As KD meals and snacks were portion-controlled, patients may also have consumed fewer calories than usual.

It should also be noted that this setting did not allow for complete control over the dietary intervention, as participants were permitted to leave the unit on weekends, and while on the unit, could interact with nonparticipant patients who were served standard fare. As this was not a metabolic research ward, urine ketone monitoring (which can be helpful in assessing dietary adherence) was burdensome for busy staff, and therefore was conducted only once per patient. However, weight loss is another piece of evidence that patients were adherent to the dietary program, as most overweight or obese individuals will lose weight on a KD (24), and clinically meaningful weight loss in people with serious mental illness is otherwise unexpected, even when lifestyle interventions aimed at weight loss are implemented (62). All but one patient lost weight including 96% of those who were taking antipsychotics, and nearly half achieved clinically significant weight loss [defined as ≥5% reduction in body weight (34)]. This welcome outcome alone makes a compelling case for the implementation of the KD in people who are taking antipsychotic medications, whether or not psychiatric symptoms improve in response to the KD, as counteracting antipsychotic-induced weight gain is extremely difficult (63).

Whether weight loss alone could lead to a reduction in symptoms of depression, bipolar disorder (64), and/or psychosis remains a largely unanswered question. In people with type 2 diabetes treated with a KD, improvement in depression symptoms was not correlated with weight loss (38). While studies find that people with clinical depression who have undergone bariatric surgery report fewer depression symptoms 6 months or more following the procedure, weight change appears to be an unreliable predictor of improvements in mood (65). We are unaware of any studies evaluating the impact of weight loss alone on symptoms of bipolar or psychotic illnesses.

### Practical Considerations

Ketogenic diet protocols exist on a spectrum ranging from the “classic” KD originally used to treat medication-refractory pediatric epilepsy (90% fat, 6% protein, 4% carbohydrate) to modified KDs which allow for higher percentages of protein, to modified Atkins diets (66), in which protein may be eaten to satiety (67). The protocol Dr. Danan chose to adapt for use with his patients falls into this last category, although protein intake was limited in this setting to 15–20% of daily calories at least 6 days per week. One advantage of this more liberal approach is that precision control of macronutrient ratios is not required, easing the logistics of both administration and adherence.

In addition to rare metabolic disorders typically diagnosed in childhood, there are several health conditions considered by many to be absolute contraindications to initiating KDs in adults; these include acute pancreatitis, nephrolithiasis, renal failure, liver failure, congestive heart failure, anorexia nervosa, and concurrent use of SGLT2 inhibitors.

A well-formulated KD can quickly and effectively lower levels of blood glucose, insulin, and blood pressure—all considered clinically desirable goals in many patients, particularly those with metabolic syndrome. However, these generally beneficial physiological adaptations to the KD necessitate careful medication management. Medications that lower blood glucose (such as insulin, sulfonylureas, and meglitinides) and medications that lower blood pressure (such as diuretics and ACE inhibitors) warrant diligent monitoring as some may need to be reduced or even discontinued as early as day 1 of KD initiation to minimize the risk of hypoglycemic, hypotensive, and hypovolemic events (68, 69).

We are unaware of published research on the potential interactions between ketogenic diets and psychotropic medications with the notable exception of anticonvulsants. Studies of patients with epilepsy treated with a KD find that anticonvulsant levels can change in response to the diet. Although these changes are typically small and clinically inconsequential, valproate levels can fall significantly (70). Since these agents have not yet been formally studied in people with psychiatric conditions, it would be prudent to monitor blood levels of all anticonvulsants as well as of any other psychiatric medications for which changes in blood level may be of clinical importance, such as lithium. With these cautions noted, for most adult patients, the potential benefits of simple, well-formulated KDs would seem to outweigh the potential risks (71).

As clinical response to the KD in this patient cohort typically occurred within 3 weeks or less, it should be noted that the highly variable and, in some cases, lengthy duration of hospitalization in this cohort was not related to the KD but largely to socioeconomic factors. In France, inpatient psychiatric care is cost-free for patients, bed availability is high, and hospitalization criteria are liberal, therefore stays lasting many months are commonplace. Given that this iteration of the KD was generally well tolerated and psychiatrically safe even in the complex cases that comprise this series, hospitalization may not be necessary so long as proper medical supervision is provided, particularly around medication management.

Key to the successful transition to a KD in any population is dietary education and support, and likely particularly important in this population was the provision of prepared meals and snacks, as depression, psychosis, and other serious psychiatric symptoms may otherwise have made the logistics of adopting and adhering to the KD (or any new diet) formidable. It is conceivable that a thoughtfully designed intensive outpatient program could provide the necessary structure and support for patients with serious mental illness to transition to a KD, and it is encouraging to see that nearly half of patients in this group managed to continue following a KD after discharge.

### Future Research Considerations

Our observations warrant further research into the potential of the KD to improve the lives of people with mental illness. Many unanswered questions remain in this nascent field that carefully designed, adequately powered, randomized controlled trials—whether conducted in inpatient, outpatient, or virtual settings—could begin to address. As serum beta-hydroxybutyrate measurements are currently the most reliable method of assessing metabolic response to the KD, daily monitoring of this metric would help clinicians and patients understand whether the degree and consistency of ketosis are important to clinical outcomes.

## Conclusion

In this retrospective analysis of clinical care, which to our knowledge represents the largest number of people with serious mental illness treated with a KD in a hospital setting thus far, we found that the KD was feasible, safe, well-tolerated, and associated with considerable improvements in mental health symptoms as well as in multiple markers of metabolic health. While more rigorous research is needed to confirm the association between the KD and improved mental health outcomes, these findings indicate that medically supervised carbohydrate restriction is a simple, safe, universally accessible intervention well worth considering as an adjunctive strategy in the treatment of serious mood and psychotic illnesses.

## Data Availability Statement

The raw data supporting the conclusions of this article will be made available by the authors, without undue reservation.

## Ethics Statement

This treatment program was approved by the Clinique du Castelviel (Castelmarou, France) Administration and Ethics Review. The patients/participants provided their written informed consent to participate in this study.

## Author Contributions

AD conceived of and implemented the intervention and generated clinical data and observations. GE conducted the literature review and wrote the manuscript. EW and LS performed statistical analyses. All authors contributed to the article and approved the final manuscript.

## Conflict of Interest

EW received consulting fees from Hill United Health and founded Adapt Your Life, Inc. (equity interest)—both companies founded on low-carbohydrate-diet principles—and received royalties for books that recommend a carbohydrate-restricted diet. GE reports stock options in DietDoctor.com, a company founded on low-carbohydrate principles. The remaining authors declare that the research was conducted in the absence of any commercial or financial relationships that could be construed as a potential conflict of interest.

## Publisher's Note

All claims expressed in this article are solely those of the authors and do not necessarily represent those of their affiliated organizations, or those of the publisher, the editors and the reviewers. Any product that may be evaluated in this article, or claim that may be made by its manufacturer, is not guaranteed or endorsed by the publisher.
