# Supplementary material for: The Ketogenic Diet for Refractory Mental Illness: A Retrospective Analysis of 31 Inpatients
Source: Front Psychiatry. 2022 Jul 6;13:951376. doi: 10.3389/fpsyt.2022.951376 (PMC9299263; doi:10.3389/fpsyt.2022.951376)
Supplement: Supplementary file 1 [file Data_Sheet_1.PDF]

Supplement: Ketogenic Diet Protocol

The ketogenic dietary protocol implemented was adapted from Dr. Eric Westman's modified Atkins induction phase diet,[1] which limits total carbohydrate intake to 20 grams per day by adhering to the guidelines below. The hospital dietitian used these guidelines to create a menu plan consisting of three meals per day that were served to all participants at least six days per week. The intervention menu deviated from the guidelines below in only one important respect, which was that quantities of meat, seafood, poultry, and eggs were limited rather than consumed to satiety, resulting in a plan with a macronutrient composition of 15–20% protein, 75–80% fat, and 5% carbohydrate.

1. Eat to satiety: meat, seafood, poultry, and eggs—including their natural fats, free of starchy coating [*Note: the meal plan used in this intervention limited daily protein intake to 15–20% of total calories*].
2. Eat 2 cups of salad vegetables per day (measured raw).
   Permitted vegetables include all leafy greens, arugula, bok choy, cabbage, chives, endive, radishes, scallions, and watercress.
3. Eat 1 cup of cooked vegetables per day (measured cooked).
   Permitted vegetables include artichokes, asparagus, broccoli, Brussels sprouts, cauliflower, celery, cucumber, eggplant, green beans, jicama, leeks, mushrooms, okra, olives, onions, peppers, pumpkin, shallots, snow peas, bean sprouts, alfalfa sprouts, sugar snap peas, summer squash, tomatoes, rhubarb, wax beans, and zucchini.
4. Permitted snack foods: pate, ham, chorizo, salami, hard-boiled eggs, sugar-free gelatin

Foods Allowed in Limited Quantities:

- Cheese: up to 100 g per day
- Creams and oils: 2 to 8 tablespoons per day, depending on weight loss goals
- Mayonnaise: 2 to 3 tablespoons per day
- Lemon juice: up to 4 teaspoons per day
- Avocado: ½ to 1 per day
- 85% dark chocolate: 2 squares per day
- Soy sauce: up to 2 tablespoons per day

Patients were advised that, during the first few days, some people will experience "keto flu" (headaches, fatigue, sleeplessness, body aches, brain fog). To reduce the risk of this happening, they were advised to drink plenty of fluids when thirsty and to consume broth twice a day (except in cases of hypertension or heart failure).

---

1. Yancy WS Jr, Olsen MK, Guyton JR, Bakst RP, Westman EC. A low-carbohydrate, ketogenic diet versus a low-fat diet to treat obesity and hyperlipidemia: a randomized, controlled trial. Ann Intern Med (2004) 140(10):769-77. doi: 10.7326/0003-4819-140-10-200405180-00006

Meal Plan:

Breakfast:

Coffee with 2 to 3 tablespoons of coconut oil OR 40–60 g of butter
1 boiled egg OR 30g of nuts OR 30 g of cheese

Lunch (main meal):

Appetizer (one of the following):

- 1 egg with 20-30 g of mayonnaise
- Half an avocado
- 2 sardines
- 2 mackerel
- 1 slice of ham
- 3 small slices of dry salami

Main dish:

100–150 g of meat, seafood, or poultry
200–250 g of cooked vegetables from the permitted list (for example cauliflower, eggplant, or ratatouille) with 2 to 3 tablespoons of olive oil

Dessert (one of the following):

- 50 g of cheese
- 2 squares of 85% dark chocolate
- 30 g of nuts

Dinner (examples):

2-egg omelet with ham or cheese
OR
2 fried eggs over ratatouille with 1 to 2 slices of chorizo
AND
Chicken soup with 2 to 3 tablespoons of olive oil OR 30 g of cheese OR 2 tablespoons of sour cream

Snacks:

Participants were each given 1 snack box per day containing:
30 g nuts, 21 g cheese, 2 squares of 85% dark chocolate, and one of the following:

- 1 to 2 hard-boiled eggs
- 1 egg plus ~100 g sardines or mackerel
- 3 slices of salami
- 2 slices of ham, 2 slices of cheese, and 1 small cucumber
